# Benthic molluscan macrofauna structure in heavily trawled sediments (Thermaikos Gulf, North Aegean Sea): spatiotemporal patterns

**DOI:** 10.1186/2241-5793-21-10

**Published:** 2014-06-10

**Authors:** Charalampos Dimitriadis, Drosos Koutsoubas, Zoi Garyfalou, Anastasios Tselepides

**Affiliations:** Department of Marine Sciences, Faculty of Environment, University of the Aegean, 81000 Mytilene Lesvos Island, Greece; Department of Maritime Studies, University of Piraeus, G. Lambraki 21 & Distomou, 18534 Piraeus, Greece; National Marine Park of Zakynthos, El. Venizelou 1, 29100 Zakynthos Island, Greece

**Keywords:** Bottom trawling, Sediment profile, Thermaikos gulf, Macrofauna

## Abstract

**Background:**

Spatio-temporal patterns on benthic molluscan macrofauna structure and function (feeding guilds) were investigated in a commercial fishing ground in Thermaikos Gulf (N Aegean Sea). Fishery management measures in this area include a trawling period of 8 months per year (October to May). Macrofauna samples were collected before and after 30 and 120 days of the commencement of the trawling period (temporal axis) along a southward transect (spatial axis) and down through the sediment profile (vertical axis).

**Results:**

Main results revealed no recognizable changes in community diversity and structure at temporal scales. This finding can probably be attributed to the fact that the examined communities are subjected to continuous disturbances deriving from multiple natural and anthropogenic stressors acting simultaneously in Thermaikos Gulf. Molluscan assemblages were already stressed before the commencement of the trawling period, indicating that the time period in which bottom trawling is prohibited is not sufficient for the recovery of benthic communities. Significant shifts in the trophic structure of molluscan assemblages were also detected. The direct mortality of herbivorous species and the loss of filter feeding organisms may be attributed to the passage of the fishing gear and to sediment re-suspension, respectively. Trawling disturbance may have created the observed vertical patterns of the community structure since hauling induces profound changes in the geochemical profile of the sediment.

**Conclusions:**

Our findings sustained the notion that bottom trawling, alongside with other types of human induced stressors, can have considerable effects on the structure and function of the benthic domain. Therefore, our results highlighted the need of an Ecosystem Based Fishery Management (EBFM) perspective in Thermaikos Gulf to ensure both fisheries and ecosystem sustainability.

**Electronic supplementary material:**

The online version of this article (doi:10.1186/2241-5793-21-10) contains supplementary material, which is available to authorized users.

## Background

In the last decades, there has been growing evidence of the wide effects of bottom trawling on marine ecosystems [1–4]. Nowadays, it is well known that physical disturbance caused by bottom trawling can be classified as one of the most important sources of human induced disturbance to soft-sediment benthic communities and habitats [5–7]. Both experimental and field studies have shown that bottom trawling modifies seabed morphology and complexity, changes the community structure of the resident biota and affects benthic production and functionality [1, 6, 8–10]. These alterations of the benthic ecosystem could in return induce secondary impacts on many commercially exploited fish species affecting total production [4].

Macrofauna is frequently used to detect bottom trawling disturbance because it is relatively easy to sample and process, is directly affected by the passage of the fishing gear as species are directly killed or damaged, and it also provides information about habitat structure [2]. In this respect, bottom trawling effects are known to be harsher for species with hard shells, larger body sizes and slow life histories rather than for flexible species with smaller body sizes ([6, 11, 12] but also see [13]). Therefore, it is reasonable to expect that larger bivalves will suffer higher mortality rates from trawling while smaller bivalves, gastropods and polychaetes will present lower mortality since lighter organisms are pushed aside by the pressure wave in front of the fishing gear [14]. As a result, in intensively trawled sediments small infaunal species are expected to proliferate, since they usually exhibit higher resilience after disturbance events, whereas larger epifaunal organisms are expected to be absent [14, 15].

While many field studies on benthic fauna response to bottom trawling have been conducted in northern European, little is known for Eastern Mediterranean waters [16, 17] despite the fact that it is characterized by unique attributes [18, 19], rich benthic biota [20], intense fishing pressure and overfished stocks [21, 22]. Thermaikos Gulf (N. Aegean Sea, Eastern Mediterranean) is characterized by increased productivity, and by an extended self (180 km long × 55 km wide) with smooth bathymetry, which is mainly comprised of soft sediments [23] and references therein]. In this respect, it is an ideal area for bottom trawling, constituting one of the most important fishing grounds in Greek waters [24]. According to Greek law (Presidential Decree 189/1978) the trawling season spans from October to the end of May (8 months per year), whereas trawling activity is permanently banned in the inner part of Thermaikos Gulf (Thessaloniki Bay) in an effort to protect the fish stocks.

The present study aims to address the structure and function (feeding guilds) of macrobenthic molluscan assemblages in a heavily trawled fishing ground of the Eastern Mediterranean, Thermaikos Gulf (N Aegean Sea), along spatiotemporal axes, down through the sediment profiles.

## Results and discussion

### Spatial and temporal patterns in molluscan diversity

A total of 4410 organisms belonging to 74 Molluscan species were recorded in the study area. Gastropoda was the most dominant class, in terms of species number (64%), followed by Bivalvia (32%), Scaphopoda (2%) and Aplacophora (2%). Considering their zoogeographic affinity, most of the collected species were of Atlanto- Mediterranean origin (60%), whereas Boreal and Endemic species presented much lower numbers (26% and 14%, respectively). Infaunal organisms dominate constituting 70% of the collected species. In terms of numerical dominance, the bivalve *Corbula gibba* accounted for 26.7% of the total abundance, whereas the gastropod *Turritella communis* and the bivalves *Kurtiella bidentata* and *Thyasira biplicata* accounted for 13.6%, 13.1% and 7.7% of total abundance, respectively.

**Figure 1:**
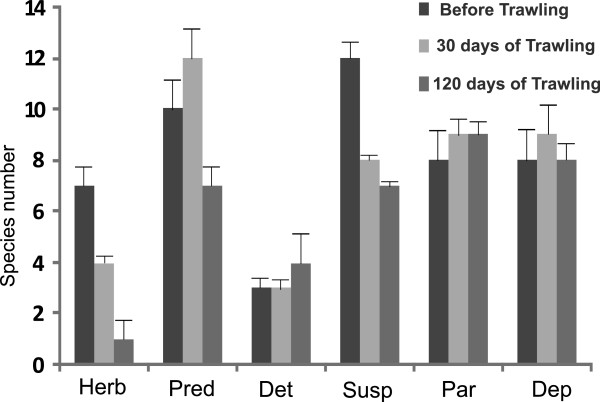
**Feeding guilds’ average species number.** Average of species number of each feeding guild before (■) and after 30 (▧) and 120 days (▦) of trawling activity (Herb = Herbivores, Pred = predators, Det = detritus feeders, Susp = suspension feeders, Par = parasites, Dep = deposit feeders) (Error bars represent standard error of average).

Predators (27%) were the dominant feeding guild, in terms of species number (Figure 1), followed by parasites (23%), suspension (20%) and deposit feeders (16%). The rest of the feeding guilds (herbivores and detritus feeders) accounted for the remaining 14%. Though the spatial variation of the allocation of species into feeding guilds within each sampling period was not significant (Man-Whitney test results, *p* > 0.05 in all cases). Herbivores’ diversity significantly decreased (Mann–Whitney test results, *p* < 0.05) at both after-trawling periods. A similar pattern was recorded for suspension feeders (Mann–Whitney test results, *p* < 0.05), whereas, significantly lower number of predatory species was detected only during the second after-trawling period.

Hierarchical ANOVA model (two-way design) results regarding the observed spatial and temporal patterns of molluscan fauna descriptors’ variation are presented in Table 1. Our findings primarily suggested that temporal variation (between different sampling periods) of descriptors’ values was not significant (*p* > 0.05 in all cases). However, spatial variability seemed to significantly account for the observed differences of abundance and diversity indices values. Pair-wise comparisons revealed that abundance values in station IP-38 were significantly higher in comparison to the rest of the sampling stations during September sampling period as well to the ones measured at station IP-10 during October sampling period (*p* < 0.05). When N1 index was examined, station IP-38 presented significantly higher values than the ones recorded in station IP-10 during all sampling periods (*p* < 0.05 in all cases). Evenness of the molluscan fauna (N21 index) varied considerably along the sampling transect since all the pair-wise comparisons between the sampling stations within each sampling period revealed statistically significant differences (*p* < 0.05 in all cases).

**Table 1:** **Summary of results of two-way ANOVA considering biological descriptors values**

Source of variation	Species number (S)	Abundance (N)	N1	N21
Sampling period	df = 2	df = 2	df = 2	df = 2
	MS = 0.015	MS = 0.444	MS = 0.035	MS = 0.002
	F = 1.447	F = 2.244	F = 0.860	F = 1.876
	*p* = 0.307	*p* = 0.187	*p* = 0.469	*p* = 0.233
Station (Sampling period)	df = 6	df = 6	df = 6	df = 6
	MS = 0.010	MS = 0.198	MS = 0.041	MS = 0.001
	F = 0.681	F = 5.891	F = 2.814	F = 3.143
	*p* = 0.667	*p* = 0.002*	*p* = 0.041*	*p* = 0.027*

Summary of results of two-way ANOVA considering species number (S), species abundance (N) and Hill’s diversity indices (N1 and N21) for factors sampling period (temporal effect-three levels: prior to trawling period, 30 and 120 days after intense bottom trawling period) and station (spatial effect-three levels: station IP-10, IP-17 and IP-38) (asterisk denotes statistically significant results).

### Molluscan community structure

**Figure 2:**
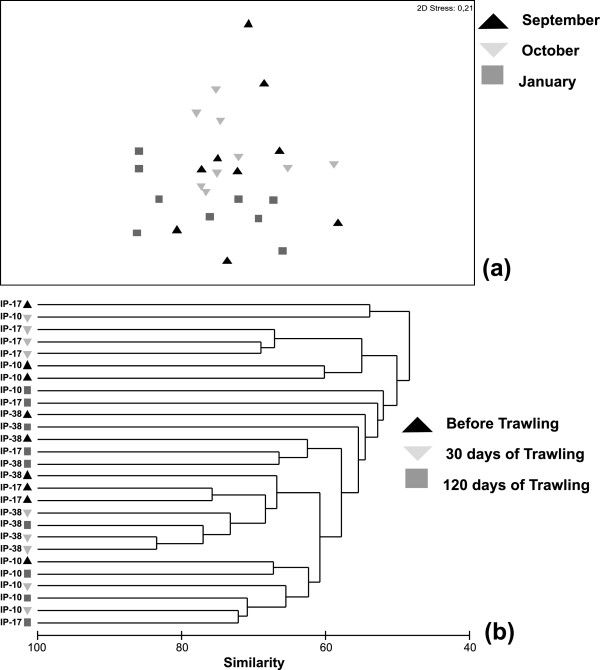
**Multivariate patterns of community structure. (a)** MDS ordination plot and **(b)** Cluster analysis of the sampling stations before the opening of the trawling season (September) as well as 30 (October) and 120 days (January) after.

Multivariate analyses (Figure 2 & Table 2) did not reveal any consistent pattern in community structure with respect to the different sampling periods (temporal effect) and therefore there were no recognizable changes in community structure before and after 30 and 120 days the initiation of the trawling period. Conversely, spatial variation seemed to significantly account for the observed differences in community structure (PERMANOVA results, Table 2). Pair-wise a posteriori comparisons revealed significant modifications of community structure between all the sampling stations during October as well as between stations IP-10 and IP-38 during February (*p* < 0.05).

**Table 2:** **Two-way PERMONAVA results regarding community structure**

Source of variation	df	SS	MS	Pseudo-F	***p***
Sampling period	2	3792.8813	1896.44061	1.0132	0.5024
Station (Sampling period)	6	11230.8464	1871.8077	1.9420	0.0001*
Residual	18	37962.2111	790.8794		
Total	26	191573.6451			

Summary of results of two-way PERMONAVA with respect to the temporal (three levels: prior to trawling period, 30 and 120 days after intense bottom trawling period) and spatial (three levels: station IP-10, IP-17 and IP-38) variation components of benthic community structure (asterisk denotes statistically significant results).

### Molluscan diversity across sediment profiles

Hierarchical ANOVA results (three-way design) with respect to the spatial and temporal variation of biological descriptors values (species number, abundance, diversity indices) along the sediment profiles are presented in Table 3. All the examined descriptors presented a common pattern of variation down through the sediment profiles. This pattern initially consisted of a steep decline in descriptors values at the transition from the surface sediment (0–5 cm depth) to the subjacent sediment layer (5–10 cm) (pair-wise comparisons, *p* < 0.05 in all cases). The initial steep decline was subsequently followed by a milder but still significant further decline of descriptors values deeper in the sediment (10–20 cm) (pair-wise comparisons *p* < 0.05 in all cases). Spatial variation component didn’t contribute significantly to the observed variability of descriptors values along sediment profiles. Temporal variance component (between sampling periods) seemed to account for the observed differences in abundance and N21 index variation for the following cases: a) between October and February sampling periods when N21 index is considered and b) between October and February as well as between September and February sampling periods when abundance is considered.

**Table 3:** **Three-way ANOVA results considering biological descriptors values**

Source of variation	Species number (S)	Abundance (N)	N1	N21
Sediment profile	df = 2	df = 2	df = 2	df = 2
	MS = 1.532	MS = 8.217	MS = 0.422	MS = 0.016
	F = 92.066	F = 43.408	F = 7.736	F = 14.272
	*p* = 0.001*	*p* = 0.001*	*p* = 0.022*	*p* = 0.005*
Station (Sediment profile)	df = 6	df = 6	df = 6	df = 6
	MS = 0.017	MS = 0.189	MS = 0.055	MS = 0.001
	F = 0.608	F = 1.160	F = 2.610	F = 1.324
	*p* = 0.721	*p* = 0.370	*p* = 0.053	*p* = 0.297
Sampling period (Station-Sediment profile)	df = 18	df = 18	df = 18	df = 18
	MS = 0.027	MS = 0.163	MS = 0.021	MS = 0.001
	F = 1.417	F = 3.489	F = 1.141	F = 2.794
	*p* = 0.162	*p* = 0.001*	*p* = 0.342	*p* = 0.002*

Summary of results of three-way ANOVA considering species number (S), species abundance (N) and Hill’s diversity indices (N1 and N21) for factors sediment profile (effect of different sediment layers-three levels: 0–5 cm, 5–10 cm and 10–20 cm down through the sediment), station (spatial effect-three levels: station IP-10, IP-17 and IP-38) and sampling period (temporal effect-three levels: prior to trawling period, 30 and 120 days after intense bottom trawling period) (asterisk denotes statistically significant results).

### Macrofauna structure along sediment profiles

**Figure 3:**
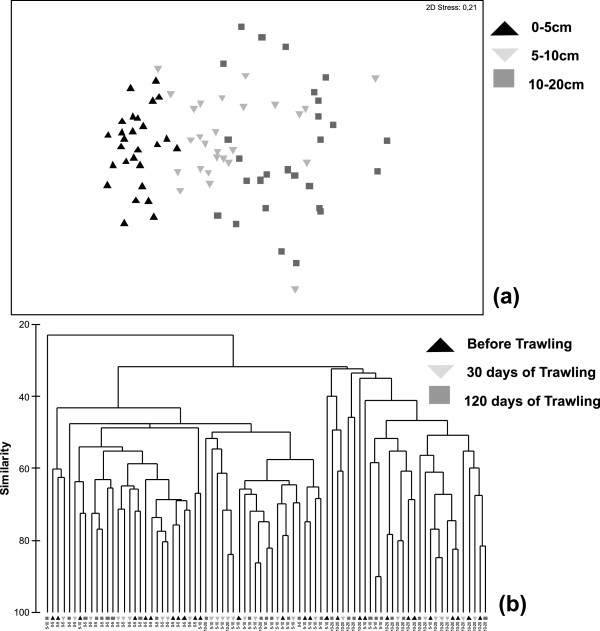
**Community structure down through the sediment profiles. (a)** MDS ordination plot for the different sediment layers (0–5 (**▲**), 5–10 (**▽**), 10–20 cm (**▧)** depth) and **(b)** Cluster analysis of the different sediment layers before (**▲**) and after 30 **▽ and 120 days** (▧) the opening of the trawling period.

Results of MDS ordination and Cluster analysis (Figure 3) demonstrated that molluscan community structure at the top 5 cm of the sediment was clearly distinguishable from the one observed deeper in the sediment. Similarly, the latter analyses also suggested the presence of different community structure of molluscan fauna between sediment layers of 5–10 cm and 10–20 cm. PERMANOVA results considering the spatial and temporal effect in community structure down though the different sediment layers are presented in Table 4. Significant changes of community structure were detected between the different sediment layers whereas Pair-wise a posteriori comparisons suggested that the observed differences in community structure were mainly attributed to the comparison of the successive sediment layers (0–5 vs 5–10, 5–10 vs 10–20 cm) down through the sediment profile (*p* < 0.05 in all cases). Spatial variation seemed to significantly account for the observed differences in the vertical community structure along the sediment mainly as a result of the comparison between station IP-38 with the rest of the sampling stations (a posteriori pair-wise comparison; *p* < 0.05). An also apparent effect of the temporal variation in community structure down through the sediment profile was detected mainly as a result of the comparison between October and February sampling periods (a posteriori pair-wise comparison; *p* < 0.05).

**Table 4:** **Three-way PERMONAVA results of community structure down through the sediment profile**

Source of variation	df	SS	MS	Pseudo-F	***p***
Sediment profile	2	43093	21547	5.1691	0.003*
Station (Sediment profile)	6	25010	4168.3	1.8651	0.002*
Sampling period (Station - Sediment profile)	18	40229	2234.9	1.4587	0.002*
Residual	54	82735	1532.1		
Total	80	191067			

Summary of results of three-way PERMONAVA of benthic community structure down through the sediment profile with respect to the temporal (three levels: prior to trawling period, 30 and 120 days after intense bottom trawling period) and spatial (three levels: station IP-10, IP-17 and IP-38) variation components (asterisk denotes statistically significant results).

### Diversity and community structure

Several authors have reported that the effects of trawling on benthic fauna diversity measures may be contradictory and as such they include decreases and increases in mean abundance of individual species, total abundance, species number and changes in various other metrics of diversity [6, 7]. Our results suggested that molluscan diversity (abundance, species number and diversity indices) was mostly affected at spatial (between sampling stations) rather than temporal (between sampling periods) scale. This finding conformed to the previously described north–south productivity gradient [17], and also suggests that the multiple natural and anthropogenic stressors [25] are acting simultaneously and in concert in Thermaikos Gulf. In this sense, given the increased dominance (in most sampling stations and periods) of *Corbula gibba, Turritella communis*, *Thyasira biplicata* and *Kurtiella bidentata*, which constitute species tolerant to organic matter enrichment and indicators of community instability [26, 27], it is reasonable to conclude that benthic communities experience continual disturbance effects.

Multivariate analysis also revealed a shift in spatial community structure within all sampling periods towards the innermost areas of Thermaikos Gulf, rather than a seasonal community shift. Therefore, our data indicated that there was no clear evidence of trawling effects on benthic community structure in Thermaikos Gulf. Similarly, Lampadariou *et al*. [17] did not find any pronounced effect of bottom trawling on the seasonal structure of the nematode community in Thermaikos Gulf. It is well known that Thermaikos Gulf constitutes an area subjected for decades to heavy urbanization, industrialization and resource exploitation, whereas discharges by three major rivers (Axios, Aliakmon and Pinios) and intense fishing activity are inducing significant pressures to its biota [24, 25]. Therefore, it seems that macrobenthic fauna is already adapted to the established disturbance regime and direct trawling effects are less visible [11, 14]. Finally, the time period in which bottom trawling is prohibited (from May to October) is probably not sufficient for the recovery of benthic communities in the study area. Several authors have pointed out that benthic communities’ recovery from bottom trawling impacts depends on the type of the habitat and its duration can vary from a few days up to many years [3, 7, 28].

### Feeding guilds

Predatory species were dominant in the study area. This finding seems to be in line with other studies reporting increased dominance of predatory macro- and meio-faunal species in heavily trawled areas or areas subjected to natural disturbance [9, 29, 30]. This can be attributed to the fact that predatory species probably gather in the tracks made by a trawl pass to feed on exposed and damaged or dead organisms [9, 11, 31]. Our results also suggested a significant decrease in the number of herbivore and suspension feeder species, after 30 and 120 days of intense trawling activity. It is broadly recognized that spatiotemporal variation of food availability is a major factor governing benthic communities’ structure [32]. However, chlorophyll-a concentrations did not vary notably before and after the commencement of trawling in the study area and therefore microphytobenthic biomass was comparable between the sampling periods [17, 33]. Thus, herbivores species reduction during October and January cannot be attributed to food limitation. On the contrary, several authors have stressed that epifaunal organisms are much more vulnerable in areas subjected to intense trawl fishing [2, 12]. In this sense, given that most of the herbivores were epibenthic species (i.e. *Alvania cimex*, *A. cimicoides*, *A. punctura, A. beanii, Rissoina bruguieri, Circulus striatus*), trawling disturbance in Thermaikos Gulf can possibly account for the reduction of macrobenthic herbivores.

It is well known that the passage of the fishing gear may induce re-suspension of a large amount of the sediment [3, 34]. Indeed, an enhanced re-suspension process was mainly induced by bottom trawling during our sampling periods [23]. In this respect, re-suspension of the silty sediment in the study area [17] may obstruct the ciliary feeding mechanisms of the suspension feeding bivalves [35]. Thus, this process could explain the reduced number of suspension feeders, observed after 30 and 120 days of trawling activity. The above hypothesis was also sustained by the findings of Kaiser *et al*. [7] who, after the meta-analysis of 55 publications, detected a significant negative impact of bottom trawling on suspension feeding organisms in muddy habitats. Pusceddu *et al*. [33] have also concluded that bottom trawling might have important trophodynamic consequences for benthic microbial and meiofaunal assemblages in Thermaikos Gulf. Hence our study further suggests that bottom trawling, alongside with other human induced stressors, can have a significant footprint in the trophic structure of macrofauna assemblages in Thermaikos Gulf. The latter trophic shift could in return alter biogeochemical processes associated with remineralization of organic material, regeneration of nutrients and nutrient fluxes as well as with benthic-pelagic coupling [10, 36, 37]. In this regard, bottom trawling is likely to induce negative impacts on the functioning of coastal ecosystems [9] and therefore future research on this topic is required.

### Vertical distribution of benthic fauna

Impacts of bottom trawling on the vertical distribution of benthic communities along the sediment are largely unknown despite the fact that trawling gear can penetrate for several centimeters into the sediment thus introducing significant changes to the morphology of the soft bottoms. Results of the present study suggested a steep decline of abundance, species number and diversity indices values from the surface down to a depth of 10 cm within the sediment, which was followed by a milder further decline in deeper sediment layers (10–20 cm). Community structure patterns also supported the existence of modifications with increasing sediment depth, mainly during the October and February sampling periods. It is known that in muddy bottoms the dissolved oxygen penetrates a few millimeters through a diffusive process into the sediment, whereas it can penetrate down to 10 cm or even more through macrofaunal burrows, bioturbation activity and irrigation processes [38, 39]. Among the dominant species inhabiting the deeper layers of the sediment were the suspension feeders *Turritella communis*, *Kurtiella bidentata* and *Corbula gibba*. These species can stand organic enrichment since they construct galleries to accommodate their siphons, which they protract at the surface of the sediment pumping the well oxygenated near-bottom water [35]. Hence, their presence in the deeper layers of the sediment can be considered as typical. However, the increased abundance of the detritus feeding species *Tricolia tenuis*, of the deposit-feeding species *Hyala vitrea* and *Thyasira biplicata*, of the parasite species *Odostomia unidentata* and *O. scalaris*, and of the carnivorous species *Cylichna cylidracea* and *Bela nebula* in the deeper layers of the sediment, suggested the presence of a diverse benthic fauna, which includes many functional groups. The later pattern has been correlated with a deep redox potential discontinuity in the sediment [38, 39]. It is well known that the passage of the fishing gear induce re-suspension, re-deposition and consequently re-layering of the sediment [3, 34]. So, we assume that bottom trawl passage provokes an increase in which reduced oxygen conditions are met in the sediment (due to re-suspension, re-deposition and consequently re-layering of the sediment). This in return can increase the maximum depth at which certain species could survive. Thus, our results have shown that bottom trawling disturbance could possibly increase the depth of the aerobic layer of the sediment and consequently alter the vertical community structure of benthic species. There is, however, a need for further research on the direct effects of bottom trawling in oxygen profiles of the trawled sediments with respect to benthic communities’ vertical distribution, to provide the necessary data to support the generalization of this hypothesis.

## Conclusions

Our findings sustained the notion that bottom trawling, alongside with other types of human induced stressors, can have considerable effects on the structure and function of the benthic domain (Table 5). Therefore, fishery management in Thermaikos Gulf should be redirected from the traditional single species management which is currently active in the area to an ecosystem based fishery management (EBFM) strategy [40] to sustain the health and function of the ecosystem as well as the fishing yields that it supports.

**Table 5:** **Summary table of molluscan macrofauna response to environmental variation and bottom trawling activity**

Response	Environmental variation	Trawling impact
**Faunal diversity**	Spatial patchiness	No direct effects
	Productivity gradient	
**Community structure**	Latitudinal gradient	No direct effects
**Functional attributes**	Natural and anthropogenic stressors	Dominance of predators
	Comparable microphytobenthic biomass	Loss of epibenthic herbivores
	Trawling induced sediment resuspension	Loss of filter feeders
**Faunal structure along sediment profiles**	Dissolved oxygen gradient	Alterations in structure
**Faunal functionality along sediment profiles**	Dissolved oxygen gradient	Presence of many functional groups deeper in the sediment

## Methods

### Study area and sampling design

**Figure 4:**
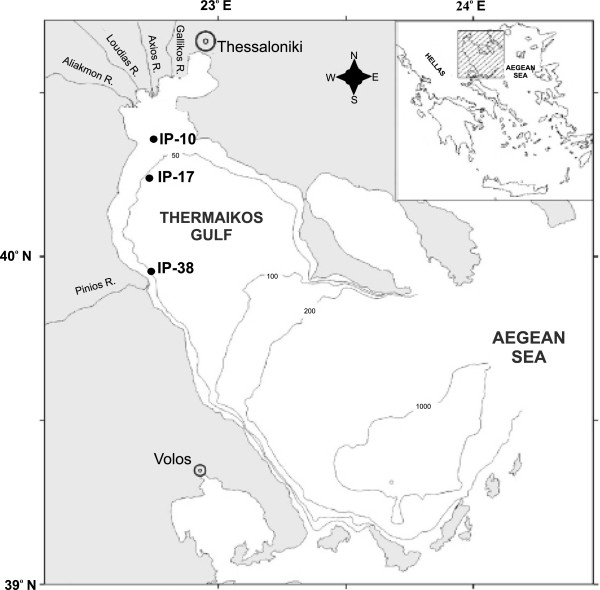
**Study area and sampling design (after Pusceddu** ***et al*** **. [**[33]**]).** Location of sampling stations (IP-10, IP-17, IP-38) across Thermaikos Gulf (N. Aegean Sea, Greece) (after Pusceddu *et al*. [33]).

Macrobenthic molluscan communities were examined at three sampling stations (Figure 4) placed across a NW-SW productivity gradient in Thermaikos Gulf, N Aegean Sea [33]. Sampling stations were located across the 50 m depth isobar since trawling activity is mostly concentrated around that depth. Sampling stations were characterized by silty sediments with mean grain size of 0.012-0.024 mm [33]. The bottom topography of Thermaikos Gulf can be considered as smooth, receiving a significant annual freshwater outflow of 10.2 × 10^6^ m^3^ from three major rivers (Aliakmon, Axios and Pinios) [17]. Trawling season, in the study area, opens at the beginning of October and ends in May, while trawl fishing is prohibited during the rest of the year. Sampling was carried out at three periods. The first one took place just before the opening of the trawling season (September 2001 - pre-trawling period, dry calm), while the second and third corresponded to one (October 2001 - initiation of trawling, pre-storming low river input period) and four (January 2002 - integrated stormy, high river input and trawling period) months after the initiation of trawling, respectively. This design was based on the before-after treatment approach to detect possible trawling impacts on molluscan communities [41]. Three replicated samples were collected at each sampling station by means of a 0.25 m^2^ USNEL boxcorer that is designed for undisturbed samples while it penetrates to a depth of 20–30 cm into the sediment [see [17] for further details]. Geochemical and physical characteristics of the sampled sediments were described by Pusceddu *et al*. [33] and Lampadariou *et al*. [17].

### Treatment of samples

The collected samples were sliced into 3 sections. The first section included the surface sediment (0–5 cm depth), the second the layer that follows just below (5–10 cm) and the remaining the deeper part of the sediment cores (10–20 cm). All samples were washed through 0.5 mm sieve, fixed in 10% formalin and kept separately with the addition of the vital stain Rose Bengal. Macrofauna was sorted into major taxonomic groups and all living Mollusca were identified to species level, counted and weighed.

### Data analyses

Data analyses involved the measurement of several biological descriptors such as species number, species abundance and the following diversity indices: Ν_1_ Hill’s index and Hill’s Evenness N21 (N2/N1) [42]. Classification of molluscs according to their feeding guilds was based on relative literature ([43, 44] and references therein) and online data bases such as the European Register of Marine Species (ERMS) [45] and the Marine Life Information Network (MARLIN) [46] (Additional file 1). Hierarchical two-way ANOVA was used to detect temporal (i.e. sampling periods, 3-level factor) and spatial (3-level factor, nested in sampling periods) variation of biological descriptors values in the study area. Hierarchical three-way ANOVA was also used to examine the variation of biological descriptors values along the sediment profiles in spatial and temporal scale. The model included the effects of the following factors: sediment profile (3 levels: 0–5 cm, 5–10 cm and 10–20 cm down through the sediment), sampling station (spatial effect) nested in sediment profile and sampling period (spatial effect) nested in sampling station and sediment profile (temporal effect). Model design was based on the recommendations of Ysebaert & Herman [47] and references therein. Prior to analysis, data were properly transformed [i.e. log(1 + x)] when normality and heterogeneity of variance were not met. Significant differences of the allocation of species into feeding guilds between sampling stations and periods were detected with the Mann–Whitney test procedure. Non metric MDS and Cluster analysis were applied for the ordination and classification of samples into groups of similar community structure, considering, as well, sediment depth profile. A two-way PERMANOVA [48] was employed to detect significant differences in benthic community structure with respect to the temporal and spatial variation components under the same model design of two-way ANOVA. A three-way PERMANOVA was applied to detect significant differences of community structure along the successive sediment layers in time (temporal effect) and space (spatial effect), under the same model design of three-way ANOVA. Multivariate analyses were based on Bray-Curtis similarity index derived by the transformed (fourth root option) species abundance data.

All calculations were performed with the use of PRIMER v6 software package [49] and SPSS v20 [50].

## Electronic supplementary material

Additional file 1: Table S1: Molluscan species assignment to their respective feeding guilds. Species list and assignment of each molluscan species to feeding guilds. (DOCX 22 KB)
