# Supplementary material for: Benthic molluscan macrofauna structure in heavily trawled sediments (Thermaikos Gulf, North Aegean Sea): spatiotemporal patterns
Source: J Biol Res (Thessalon). 2014 Jun 10;21(1):10. doi: 10.1186/2241-5793-21-10 (PMC4389299; doi:10.1186/2241-5793-21-10)
Supplement: Supplementary file 1 — Additional file 1: Table S1: Molluscan species assignment to their respective feeding guilds. Species list and assignment of each molluscan species to feeding guilds. (DOCX 22 KB) [file 40709_2013_10_MOESM1_ESM.docx]

| **Species List** | **Feeding Guild** |
| --- | --- |
| *Acanthocardia aculeata* (Linnaeus, 1758) | Suspension feeder |
| *Acanthocardia echinata* (Linnaeus, 1758) | Suspension feeder |
| *Acanthocardia spinosa* (Lightfoot, 1786) | Suspension feeder |
| *Alvania beanii* (Hanley in Thorpe, 1844) | Herbivore |
| *Alvania cimex* (Linnaeus, 1758) | Herbivore |
| *Alvania cimicoides* (Forbes, 1844) | Herbivore |
| *Alvania discors* (Allan, 1818) | Herbivore |
| *Alvania discors* (Allan, 1818) | Herbivore |
| *Alvania hispidula* (Monterosato, 1884) | Herbivore |
| *Alvania punctura* (Montagu, 1803) | Herbivore |
| *Antalis inaequicostata* (Dautzenberg, 1891) | Predator |
| *Antalis vulgaris* (da Costa, 1778) | Deposit feeder |
| *Arcopagia balaustina* (Linnaeus, 1758) | Deposit feeder |
| *Bela nebula* (Montagu, 1803) | Predator |
| *Circulus striatus* (Philippi, 1836) | Herbivore |
| *Corbula gibba* (Olivi, 1792) | Suspension feeder |
| *Crenilabium exile* (Jeffreys, 1870) | Predator |
| *Ctena decussata* (O. G. Costa, 1829) | Deposit feeder |
| *Cylichna cylindracea* (Pennant, 1777) | Predator |
| *Dosinia exoleta* (Linnaeus, 1758) | Suspension feeder |
| *Eulimella acicula* (Philippi, 1836) | Parasite |
| *Eulimella cerullii* (Cossmann, 1916) | Parasite |
| *Eulimella unifasciata* (Forbes, 1844) | Parasite |
| *Euspira intricata* (Donovan, 1804) | Predator |
| *Falcidens gutturosus* (Kowalevsky, 1901) | Predator |
| *Fusinus rostratus* (Olivi, 1792) | Predator |
| *Gari costulata* (Turton, 1822) | Suspension feeder |
| *Hyala vitrea* (Montagu, 1803) | Deposit feeder |
| *Kurtiella bidentata* (Montagu, 1803) | Suspension feeder |
| *Loripes lucinalis* (Lamarck, 1818) | Deposit feeder |
| *Mactra stultorum* (Linnaeus, 1758) | Suspension feeder |
| *Mangelia attenuata* (Montagu, 1803) | Predator |
| *Mangelia payraudeauti* (Deshayes, 1835) | Predator |
| *Mangelia unifasciata* (Deshayes, 1835) | Predator |
| *Megastomia conoidea* (Brocchi, 1814) | Parasite |
| *Moerella donacina* (Linnaeus, 1758) | Deposit feeder |
| *Myrtea spinifera* (Montagu, 1803) | Deposit feeder |
| *Nassarius incrassatus* (Strøm, 1768) | Predator |
| *Nassarius pygmaeus* (Lamarck, 1822) | Predator |
| *Neolepton sulcatulum* (Jeffreys, 1859) | Deposit feeder |
| *Nucula nucleus* (Linnaeus, 1758) | Deposit feeder |
| *Obtusella macilenta* (Monterosato, 1880) | Deposit feeder |
| *Ocinebrina aciculata* (Lamarck, 1822) | Predator |
| *Odostomia acuta (*Jeffreys, 1848) | Parasite |
| *Odostomia eulimoides* (Hanley, 1844) | Parasite |
| *Odostomia lukisii* (Jeffreys, 1859) | Parasite |
| *Odostomia scalaris* (MacGillivray, 1843) | Parasite |
| *Odostomia turrita* (Hanley, 1844) | Parasite |
| *Odostomia unidentata* (Montagu, 1803) | Parasite |
| *Ondina diaphana* (Jeffreys, 1848) | Parasite |
| *Ondina sp.* (de Folin, 1870) | Parasite |
| *Ondina warreni* (Thompson, 1845) | Parasite |
| *Parvicardium exiguum* (Gmelin, 1791) | Suspension feeder |
| *Parvicardium pinnulatum* (Conrad, 1831) | Suspension feeder |
| *Parvicardium scabrum* (Philippi, 1844) | Suspension feeder |
| *Phaxas adriaticus* (Coen, 1933) | Suspension feeder |
| *Prochaetoderma raduliferum* (Kowalevsky, 1901) | Deposit feeder |
| *Raphitoma cordieri* (Payraudeau, 1826) | Predator |
| *Raphitoma pupoides* (Monterosato, 1884) | Predator |
| *Raphitoma servaini* (Locard, 1891) | Predator |
| *Ringicula auriculata* (Ménard de la Groye, 1811) | Predator |
| *Rissoina bruguieri* (Payraudeau, 1826) | Predator |
| *Setia ambigua* (Brugnone, 1873) | Detritus feeder |
| *Thracia convexa* (W. Wood, 1815) | Suspension feeder |
| *Thracia phaseolina* (Lamarck, 1818) | Suspension feeder |
| *Thracia pubescens* (Pulteney, 1799) | Suspension feeder |
| *Thyasira flexuosa* (Montagu, 1803) | Deposit feeder |
| *Tricolia tenuis* (Michaud, 1829) | Parasite |
| *Trophonopsis muricata* (Montagu, 1803) | Predator |
| *Turbonilla amoena* (Monterosato, 1878) | Parasite |
| *Turbonilla jeffreysii* (Jeffreys, 1848) | Parasite |
| *Turbonilla pumila* (Seguenza G., 1876) | Parasite |
| *Turritella communis* (Risso, 1826) | Detritus feeder |
| *Volvulella acuminata* (Bruguière, 1792) | Predator |
